# A regime-switching SIR epidemic model with a ratio-dependent incidence rate and degenerate diffusion

**DOI:** 10.1038/s41598-019-47131-6

**Published:** 2019-07-23

**Authors:** Zhongwei Cao, Xu Liu, Xiangdan Wen, Liya Liu, Li Zu

**Affiliations:** 1 Department of Applied Mathematics, Jilin University of Finance and Economics, Changchun, 130117 Jilin Province P.R. China; 2 Department of Mathematics, Yanbian University, Yanji, 133002 Jilin Province P.R. China; 3 College of Science, China University of Petroleum (East China), Qingdao, 266580 Shandong Province P.R. China; 4 College of Mathematics and Statistics, Hainan Normal University, Haikou, 571158 Hainan Province P.R. China

**Keywords:** Infectious diseases, Statistics

## Abstract

In this paper, we present a regime-switching SIR epidemic model with a ratio-dependent incidence rate and degenerate diffusion. We utilize the Markov semigroup theory to obtain the existence of a unique stable stationary distribution. We prove that the densities of the distributions of the solutions can converge in *L*^1^ to an invariant density under certain condition. Moreover, the sufficient conditions for the extinction of the disease, which means the disease will die out with probability one, are given in two cases. Meanwhile, we obtain a threshold parameter which can be utilized in identifying the stochastic extinction and persistence of the disease. Some numerical simulations are given to illustrate the analytical results.

## Introduction

Based on the pioneering research^1^, mathematical model provides effective control measures for infectious diseases and is an significant tool for analyzing the epidemiological characteristics of infectious diseases^2^. In the course of the spread of disease, the transmission function plays an important role in determining disease dynamics (see e.g.^3,4^). There are several nonlinear transmission functions proposed by authors (see e.g.^5–8^). For instance, in^5^, Capasso and Serio introduced a saturated incidence rate *g*(*I*)*S* into epidemic models, and the infectious force *g*(*I*) is a function of an infected individual that is applied in many classical disease models. Liu *et al*.^6^ proposed a general incidence rate $g(I)S=\frac{\beta {I}^{p}S}{1+\rho {I}^{q}}$, *p*, *q* > 0. Lahrouz *et al*.^7^ introduced a more generalized incidence rate $g(I)S=\frac{SI}{f(I)}$. In particular, Yuan and Li^8^ considered a ratio-dependent nonlinear incidence rate in the following form

1.1 $$g(\frac{I}{S})S=\frac{\beta {(I/S)}^{l}S}{1+\alpha {(I/S)}^{h}}=\frac{\beta {S}^{h-l+1}{I}^{l}}{{S}^{h}+\alpha {I}^{h}},\,h,l\ge 1,$$

where *α* is a parameter used to measure psychological or inhibitory effects. We note that if *α* = *h* = *l* = 1, then (1.1) becomes the frequency dependent transmission rate (or the standard incidence rate $g(\frac{I}{S})S=\frac{\beta SI}{S+I}$).

In the case of *l* = 1, Cai *et al*.^9^ obtained a ratio-dependent transmission rate $g(\frac{I}{S})S$ which takes the following form

1.2 $$g(\frac{I}{S})S=\frac{\beta I}{1+\alpha {(I/S)}^{h}}=\frac{\beta {S}^{h}I}{{S}^{h}+\alpha {I}^{h}},$$

which means that the rate of spread of the disease is approximately controlled by *βI* in the early stages of disease transmission (e.g., $\frac{I}{S}$ is small) and it is approximately controlled by $\frac{\beta }{\alpha }{(\frac{S}{I})}^{h-1}S$ in the endemic when almost everyone is infected, in other words, when $\frac{I}{S}$ is large. Thus the ratio-related incidence of transmission (1.2) takes into account crowding effects and behavioural changes during epidemics.

In this paper, we first study the following SIR epidemic model with a ratio-dependent incidence rate

1.3 $$\{\begin{array}{rcl}\frac{d{S}_{t}}{dt} & = & {\rm{\Lambda }}-\mu {S}_{t}-\frac{\beta {S}_{t}^{h}{I}_{t}}{{S}_{t}^{h}+\alpha {I}_{t}^{h}},\\ \frac{d{I}_{t}}{dt} & = & \frac{\beta {S}_{t}^{h}{I}_{t}}{{S}_{t}^{h}+\alpha {I}_{t}^{h}}-(\mu +\gamma +\varepsilon ){I}_{t},\end{array}$$

where the parameters Λ, *μ*, *β*, *α*, *γ* and *ε* are all positive constants. Due to the fact that the dynamics of compartment *R* is without influence upon the dynamics of the transmission of the disease, it was left out from the model (1.3). In model (1.3), *S*_*t*_ and *I*_*t*_ represent the number of susceptible individuals and infected individuals, respectively.The parameters in system (1.3) are explained as follows:

Λ: the influx of individuals into the susceptible;

*μ*: the natural death rate is;

*ε*: the additional death due to disease;

*β*: the disease transmission coefficient;

*γ*: the rate of recovery from infection.

In system (1.3), the basic reproduction number is ${ {\mathcal R} }_{0}=\frac{\beta }{\mu +\gamma +\varepsilon }$ which determines the disease occurs or not. If ${ {\mathcal R} }_{0} < 1$, system (1.3) has only the disease-free equilibrium ${E}_{0}=(\frac{{\rm{\Lambda }}}{\mu },0)$ which always exists and it is globally asymptotically stable in the invariant set Γ, where ${\rm{\Gamma }}=\{(S,I)\in {{\mathbb{R}}}_{+}^{2}:\frac{{\rm{\Lambda }}}{\mu +\gamma +\varepsilon }\le S+I\le \frac{{\rm{\Lambda }}}{\mu }\}$, while if ${ {\mathcal R} }_{0} > 1$, then *E*_0_ is unstable and system (1.3) has a unique positive endemic equilibrium E^*^ = (S^*^, I^*^) which is globally asymptotically stable in the region Γ, where ${S}^{\ast }=\frac{{\rm{\Lambda }}\mu }{{\rm{\Lambda }}\mu +{(\frac{{ {\mathcal R} }_{0}-1}{\alpha })}^{\frac{1}{h}}(\mu (\mu +\varepsilon )+\mu \gamma )}$, ${I}^{\ast }={(\frac{{ {\mathcal R} }_{0}-1}{\alpha })}^{\frac{1}{h}}{S}^{\ast }$.

We have a deeper understanding to the effects of the transmission coefficient *β* on the basis of these useful research about deterministic epidemic models. However, in the real world, the spread of infectious diseases are always subject to random fluctuations, it is more reasonable and practical to study the influence of random factors. Usually, environmental noise can be simply divided into white noise and colored noise. The disease transmission coefficient *β* in the SIR model is a key parameter for disease transmission, so it is interesting to assess the effect of the perturbation parameter *β* on the model. In applications we usually estimate it by an average value plus errors. Assume these errors follow a normal distribution, that is,

$$\beta \to \beta +\sigma {\dot{B}}_{t},$$

thus *βdt* → *βdt* + *σdB*_*t*_, where *σ*^2^ > 0 is the intensity of the white noise, *B*_*t*_ is a standard Brownian motion defined on a complete probability space $({\rm{\Omega }}, {\mathcal F} ,{\{{ {\mathcal F} }_{t}\}}_{t\ge 0},{\mathbb{P}})$ with a filtration ${\{{ {\mathcal F} }_{t}\}}_{t\ge 0}$ satisfying the usual conditions (see^10^). Recently, epidemic models described by stochastic differential equations have been studied by many researchers (see e.g.^9,11–13^). For instance, a stochastic SIRS epidemic model with a ratio-dependent incidence rate is proposed in^9^. The results show that the reproduction number ${ {\mathcal R} }_{0}^{S}$ can determine whether there is a unique disease-free stationary distribution or a unique local stationary distribution. In addition, they provide analysis of stochastic boundedness and permanent/extinction. A stochastic SIS epidemic model is considered by Gray and his coworkers^11^. They give the unique global positive solutions of the model and derive the conditions of persistence and extinction of the disease. Meng^12^ investigated dynamical properties of a novel nonlinear stochastic SIS epidemic model with double epidemic hypothesis. And Zhou *et al*.^13^ considered the property of ergodic stationary distribution for a stochastic SIR epidemic model.

In addition to white noise, epidemic models are also subject to colored noise, namely telegraph noise, which switches the system from one environmental state to another^14,15^. Now we add telegraph noise in order to make our model be more realistic^14,16^. Telegraph noise can be interpreted as switching between two or more environmental states, which vary with factors such as humidity and temperature^17,18^. Switching between environment states is usually memory-free, and the waiting time for the next switch follows an exponential distribution^19^. Therefore, the state transition can be modeled using a continuous time markov chain (*r*(*t*))_*t*≥0_ that takes the values in the finite state space ${\mathbb{S}}$= {1, 2, …, *N*}. Also,

$$\begin{array}{ccc}{\mathbb{P}}\{r(t+{\rm{\Delta }}t)=j|r(t)=i\} & = & \{\begin{array}{cc}{\gamma }_{ij}{\rm{\Delta }}t+o({\rm{\Delta }}t), & if\,i\ne j,\\ 1+{\gamma }_{ii}{\rm{\Delta }}t+o({\rm{\Delta }}t), & if\,i=j,\end{array}\end{array}$$

is the generator $\tilde{{\rm{\Gamma }}}={({\gamma }_{ij})}_{N\times N}$ of *r*(*t*) where Δ*t* > 0, *γ*_*ij*_ ≥ 0 is the transition rate from *i* to *j* for *i*, *j* = 1, 2, …, *N* with $j\ne i$ and ${\gamma }_{ii}=-\,\sum _{j\ne i}\,{\gamma }_{ij}$ for each *i* = 1, 2, …, *N*. Assume that the Markov chain *r*(*t*) is irreducible and independent of the Brownian motion *B*(*t*). Thus there is a unique stationary distribution *π* = (*π*_1_, *π*_2_, …, *π*_*N*_) of *r*(*t*) which satisfies $\pi \tilde{{\rm{\Gamma }}}=0$, $\sum _{i=1}^{N}\,{\pi }_{i}=1$ and *π*_*i*_ > 0 for any $i\in {\mathbb{S}}$.

Taking into account the above two disturbances into the model (1.3), the SIR epidemic model with a ratio-dependent incidence rate and regime-switching is as shown below

1.4 $$\{\begin{array}{rcl}d{S}_{t} & = & [{{\rm{\Lambda }}}_{r(t)}-{\mu }_{r(t)}{S}_{t}-\frac{{\beta }_{r(t)}{S}_{t}^{h}{I}_{t}}{{S}_{t}^{h}+{\alpha }_{r(t)}{I}_{t}^{h}}]dt-\frac{{\sigma }_{r(t)}{S}_{t}^{h}{I}_{t}}{{S}_{t}^{h}+{\alpha }_{r(t)}{I}_{t}^{h}}d{B}_{t},\\ d{I}_{t} & = & [\frac{{\beta }_{r(t)}{S}_{t}^{h}{I}_{t}}{{S}_{t}^{h}+{\alpha }_{r(t)}{I}_{t}^{h}}-({\mu }_{r(t)}+{\gamma }_{r(t)}+{\varepsilon }_{r(t)}){I}_{t}]dt+\frac{{\sigma }_{r(t)}{S}_{t}^{h}{I}_{t}}{{S}_{t}^{h}+{\alpha }_{r(t)}{I}_{t}^{h}}d{B}_{t},\end{array}$$

where ${\sigma }_{i}^{2} > 0$ ($i\in {\mathbb{S}}$) is the intensity of the colored noise. The parameters Λ_*i*_, *μ*_*i*_, *β*_*i*_, *α*_*i*_, *γ*_*i*_, *ε*_*i*_ and *σ*_*i*_ are all positive constants for any $i\in {\mathbb{S}}$. Model (1.4) is operated as follows: if *r*_0_ = *i*_0_, the model parameters obey ${{\rm{\Lambda }}}_{r(t)}={{\rm{\Lambda }}}_{{i}_{0}}$, ${\mu }_{r(t)}={\mu }_{{i}_{0}}$, ${\beta }_{r(t)}={\beta }_{{i}_{0}}$, ${\alpha }_{r(t)}={\alpha }_{{i}_{0}}$, ${\gamma }_{r(t)}={\gamma }_{{i}_{0}}$, ${\varepsilon }_{r(t)}={\varepsilon }_{{i}_{0}}$ and ${\sigma }_{r(t)}={\sigma }_{{i}_{0}}$ until time *τ*_1_ when the Markov chain jumps to *i*_1_; the system parameters will then satisfy ${{\rm{\Lambda }}}_{{i}_{1}}$, ${\mu }_{{i}_{1}}$, ${\beta }_{{i}_{1}}$, ${\alpha }_{{i}_{1}}$, ${\gamma }_{{i}_{1}}$, ${\varepsilon }_{{i}_{1}}$ and ${\sigma }_{{i}_{1}}$ from time *τ*_1_ till time *τ*_2_ when the Markov chain jumps to the next state *i*_2_. If the markov chain jumps, the system will continue to switch.

In recent years, many researchers have studied the regime-switching stochastic epidemic models especially the long-time behavior of them (see e.g.^20–22^). In these literatures, to prove the ergodicity of random systems, we must first prove the uniform ellipticity condition. However, the diffusion matrix of the model (1.4) is degenerate and there is no uniform ellipticity condition in this paper. As far as we know, there is little research in this area. Therefore, in this paper, we will focus on the asymptotic behavior of the solutions of stochastic systems (1.4). The method adopted in this paper is derived from the markov semigroup theory introduced in^23,24^ to study the long-term behavior of the stochastic predator-predator model. Based on markov semigroup theory, Lin *et al*.^25^ analyzed the long-term behavior of the distribution density of the solutions of random SIR epidemic model.

Throughout this paper, unless otherwise specified, let $({\rm{\Omega }}, {\mathcal F} ,{\{{ {\mathcal F} }_{t}\}}_{t\ge 0},{\mathbb{P}})$ be a complete probability space with a filtration ${\{{ {\mathcal F} }_{t}\}}_{t\ge 0}$ satisfying the usual conditions (i.e., it is increasing and right continuous while ${ {\mathcal F} }_{0}$ contains all ${\mathbb{P}}$-null sets). We use *A*^*T*^ to denote the transpose of a vector or matrix *A*, set $\hat{g}={{\rm{\min }}}_{i\in {\mathbb{S}}}\{{g}_{i}\}$ and $\check{g}={{\rm{\max }}}_{i\in {\mathbb{S}}}\{{g}_{i}\}$ for any vector *g* = (*g*_1_, *g*_2_, …, *g*_*N*_). Moreover, let ${{\mathbb{R}}}_{+}^{n}=\{x=({x}_{1},{x}_{2},\ldots ,{x}_{n})\in {{\mathbb{R}}}^{n}:{x}_{i} > 0,1\le i\le n\}$.

This paper is organized as follows. In Section 2, we study stochastically asymptotic stability, the existence of a unique stable stationary distribution and present sufficient conditions for extinction of the disease in two cases. Some numerical simulations are introduced to demonstrate the theoretical results in Section 3. Finally, we summarize the results of this paper. And in the Appendixes, we present some preliminaries and give the proofs of our main results.

## Stationary Distribution and Extinction

In the study of SIR deterministic model, disease eradication and stability are two of the most concerns. But for stochastic model, the equilibrium does not exist. Therefore, we can not show the persistence of the infection by proving the stability of the equilibrium. In this section, we will investigate the dynamics of the stochastic epidemic model (1.4). First, we will prove that system (1.4) has a stationary distribution, which implies the disease is recurrent. We will give the conclusion that if ${ {\mathcal R} }_{0}^{S} > 1$, the densities of the distributions of the solutions to system (1.4) can converge in *L*^1^ to an invariant density.

### Theorem 2.1.

Let (*S*_*t*_, *I*_*t*_, *r*(*t*)) be a solution of system (1.4) with any initial value (*S*_0_, *I*_0_, *r*(0)) ∈ *E* × ${\mathbb{S}}$, then for every *t* > 0 the distribution of (*S*_*t*_, *I*_*t*_, *r*(*t*)) has a density *u*(*t*, *x*, *y*, *i*). If ${ {\mathcal R} }_{0}^{S}=\frac{{\sum }_{i=1}^{N}\,{\pi }_{i}{\beta }_{i}}{{\sum }_{i=1}^{N}\,{\pi }_{i}({\mu }_{i}+{\gamma }_{i}+{\varepsilon }_{i}+\frac{{\sigma }_{i}^{2}}{2})} > 1$, then there is a unique density u_*_(*x*, *y*, *i*) such that

$$\mathop{\mathrm{lim}}\limits_{t\to \infty }\sum _{i=1}^{N}\,\mathop{\iint }\limits_{E}\,|u(t,x,y,i)-{u}_{\ast }(x,y,i)|dxdy=0.$$

The proof of Theorem 2.1 will be given in the Appendix B.

### Remark 2.1.

Theorem 2.1 show that if ${ {\mathcal R} }_{0}^{S} > 1$, there exists a unique stationary distribution *μ*(⋅,⋅) of model (1.4) which is ergodic. The results mean that the model is stochastically asymptotic stability. In a biological sense, our result means that if ${ {\mathcal R} }_{0}^{S} > 1$, the disease *I* is stochastic persistent. That is to say, the disease will prevail and persist in the population.

Next, we will investigate the stochastic extinction of the disease in model (1.4). To this end, we establish the following theorem.

### Theorem 2.2.

Let (*S*_*t*_, *I*_*t*_, *r*(*t*)) be the solution of system (1.4) with any initial value (*S*_0_, *I*_0_, *r*(0)) ∈ *E* × ${\mathbb{S}}$. If one of the following conditions holds

$$(i)\,{\bar{R}}^{\ast }=\frac{{\sum }_{i=1}^{N}\,{\pi }_{i}\frac{{\beta }_{i}^{2}}{2{\sigma }_{i}^{2}}}{{\sum }_{i=1}^{N}\,{\pi }_{i}({\mu }_{i}+{\gamma }_{i}+{\varepsilon }_{i})} < 1;$$

$$(ii)\,{\sigma }_{i}^{2}\le {\beta }_{i}\,{\rm{for}}\,{\rm{any}}\,i\in {\mathbb{S}}\,{\rm{and}}\,{ {\mathcal R} }_{0}^{S} < 1,$$

where ${ {\mathcal R} }_{0}^{S}$ is the same as it in Theorem 2.1, then the disease *I* goes extinct with probability one, i.e.,

$$\mathop{\mathrm{lim}}\limits_{t\to \infty }I(t)=0\,{\rm{a}}.{\rm{s}}.$$

The mathematical proof of Theorem 2.1 can be found in the Appendix C.

### Remark 2.2.

From the condition (ii) in Theorem 2.2, we can find that if ${ {\mathcal R} }_{0}^{S} < 1$ and the noises are not large, then the disease *I* dies out a.s. In addition, we notice that the expression of R in theorem 2.1 is significantly different from the basic representation number ${ {\mathcal R} }_{0}^{S}$ of the model (1.3), which is less than ${ {\mathcal R} }_{0}$. In other words, the extinction condition of *I* in system (1.4) is much weaker than the corresponding deterministic model (1.3).

### Remark 2.3.

From Theorems 2.1 and 2.2, we can conclude that the stochastic reproduction number ${ {\mathcal R} }_{0}^{S}$ can be considered as a threshold to govern the stochastic dynamics of system (1.4): if ${ {\mathcal R} }_{0}^{S} < 1$ and ${\sigma }_{i}^{2}\le {\beta }_{i}$ for any $i\in {\mathbb{S}}$, the disease *I* dies out with probability one; while if ${ {\mathcal R} }_{0}^{S} > 1$, there is a unique stationary distribution *μ*(⋅,⋅) of model (1.4) which is ergodic. It means that the disease *I* is persistent stochastically. Moreover, from the condition (i) in Theorem 2.2, we can derive that large noises can inhibit the outbreak of disease.

## Numerical Experiments

In this part, Milstein’s high Order Method^26^ was used to verify the theoretical results we obtained.

### Example 3.1.

Let’s think about *N* = 2. Let the generator $\tilde{{\rm{\Gamma }}}={({\gamma }_{ij})}_{N\times N}$ of the markov chain be

$$\tilde{{\rm{\Gamma }}}=[\begin{array}{cc}-0.2 & 0.2\\ 0.8 & -0.8\end{array}],$$

where *γ*_*ij*_ is the right-continuous markov chain at the value of ${\mathbb{S}}$ = {1, 2}. By solving the linear Equation $\pi \tilde{{\rm{\Gamma }}}=0$, we obtain the unique stationary (probability) distribution

$$\pi =({\pi }_{1},{\pi }_{2})=(\frac{4}{5},\frac{1}{5}).$$

Set the parameter values in system ((1.4) as follows

$$\begin{array}{c}{{\rm{\Lambda }}}_{1}=0.3,{\mu }_{1}=0.1,{\beta }_{1}=0.8,{\alpha }_{1}=0.2,{\gamma }_{1}=0.3,{\varepsilon }_{1}=0.1,\\ \,{{\rm{\Lambda }}}_{2}=0.5,{\mu }_{2}=0.2,{\beta }_{2}=0.6,{\alpha }_{2}=0.1,{\gamma }_{2}=0.2,{\varepsilon }_{2}=0.2,h=\mathrm{0.002.}\end{array}$$

**Figure 1:**
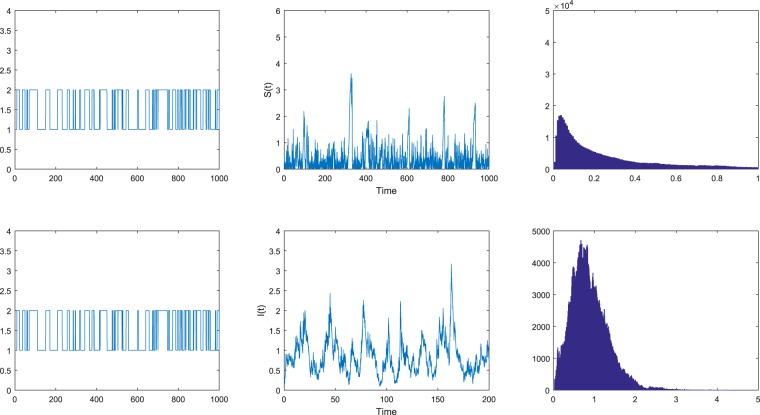
The solution (*S*_*t*_, *I*_*t*_, *r*(*t*)) of model (1.4) is ergodic. The figures on the left are the Markov chain, and the figures on the right are the probability density functions of model (1.4) with the initial value (*S*_0_, *I*_0_) = (0.3, 0.2) for *i* ∈ ${\mathbb{S}}$ = {1, 2}. The intensities of the noises are chosen as ${\sigma }_{1}^{2}=0.4$, ${\sigma }_{2}^{2}=0.2$.

**Figure 2:**
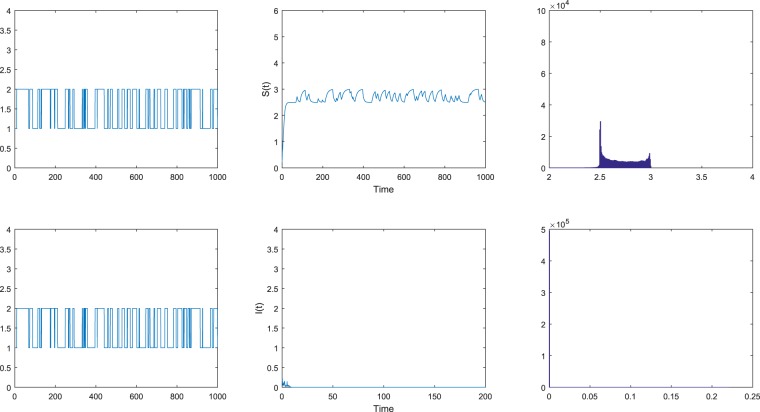
The disease *I* goes to extinction exponentially with probability one. The left figures are the Markov chain while the right figures are the probability density functions of model (1.4) with the initial value (*S*_0_, *I*_0_) = (0.3, 0.2) for *i* ∈ ${\mathbb{S}}$ = {1, 2}. The intensities of the noises are chosen as ${\sigma }_{1}^{2}=0.8$, ${\sigma }_{2}^{2}=0.6$.

**Figure 3:**
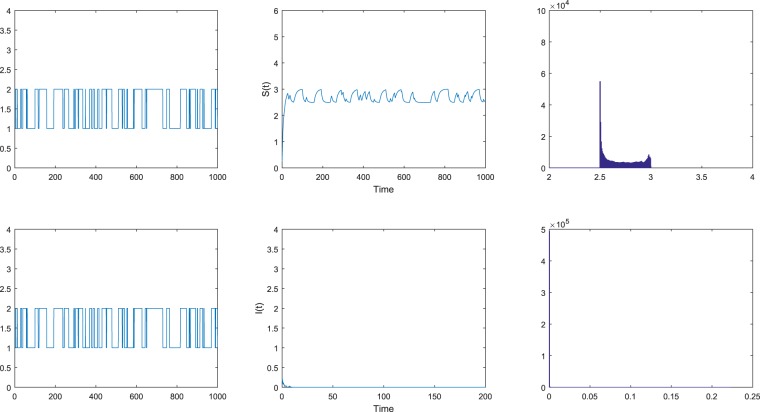
The disease *I* goes extinct with probability one. The left figures are the Markov chain and the right figures are the probability density functions of model (1.4) with the initial value (*S*_0_, *I*_0_) = (0.3, 0.2) for *i* ∈ ${\mathbb{S}}$ = {1, 2}. The intensities of the noises are chosen as ${\sigma }_{1}^{2}=0.4$, ${\sigma }_{2}^{2}=0.4$.

The values of ${\sigma }_{1}^{2}$ and ${\sigma }_{2}^{2}$ are different in Figs 1–3 while other conditions are the same. By Matlab software, we simulate the solution of system (1.4) with the three sets of white noise value, respectively. The dynamics for the two populations (S and I) are obtained for the three different data sets of white noise intensity at t = 1000.

Case (a). We select the intensities of the noises ${\sigma }_{1}^{2}=0.4$, ${\sigma }_{2}^{2}=0.2$ in Fig. 1. Then by a simple computation, we have

$${ {\mathcal R} }_{0}^{S}=\frac{{\sum }_{i=1}^{2}\,{\pi }_{i}{\beta }_{i}}{{\sum }_{i=1}^{2}\,{\pi }_{i}({\mu }_{i}+{\gamma }_{i}+{\varepsilon }_{i}+\frac{{\sigma }_{i}^{2}}{2})}\approx 1.086 > 1.$$

Hence the condition in Theorem 2.1 holds. By virtue of Theorem 2.1, we can derive that there is a unique ergodic stationary distribution *μ*(⋅,⋅) of system (1.4). See Fig. 1. In addition, it is clear from Fig. 1 that disease *I* is stochastic persistent in the mean a.s.

Case (b). In Fig. 2, we select the intensities of the noises ${\sigma }_{1}^{2}=0.8$, ${\sigma }_{2}^{2}=0.6$. We get

$${\bar{R}}^{\ast }=\frac{{\sum }_{i=1}^{N}\,{\pi }_{i}\frac{{\beta }_{i}^{2}}{2{\sigma }_{i}^{2}}}{{\sum }_{i=1}^{N}\,{\pi }_{i}({\mu }_{i}+{\gamma }_{i}+{\varepsilon }_{i})}\approx 0.731 < 1.$$

by direct calculation. Thus the condition (i) in Theorem 2.2 holds. According to the condition (i) in Theorem 2.2, we obtain that the disease *I* goes extinct with probability one. We can see this from Fig. 2.

Case (c). The intensities of the noises are chosen as ${\sigma }_{1}^{2}=0.4$, ${\sigma }_{2}^{2}=0.4$ in Fig. 3. Then direct calculation leads to

$${ {\mathcal R} }_{0}^{S}=\frac{{\sum }_{i=1}^{2}\,{\pi }_{i}{\beta }_{i}}{{\sum }_{i=1}^{2}\,{\pi }_{i}({\mu }_{i}+{\gamma }_{i}+{\varepsilon }_{i}+\frac{{\sigma }_{i}^{2}}{2})}=0.76 < 1.$$

That is to say, the condition (ii) in Theorem 2.2 is satisfied. According to the condition (ii) in Theorem 2.2, it can be seen that the disease *I* goes to extinction with probability one. We can see this phenomenon in Fig. 3.

## Conclusion

The long-time behavior of a regime-switching SIR epidemic model with a ratio-dependent incidence rate and degenerate diffusion are observed in this paper. The existence of a unique stable stationary distribution is obtained by using markov semigroup theory. It is proved that the distribution density of solutions converges to a invariant density in *L*^1^ under the condition of ${ {\mathcal R} }_{0}^{S} > 1$. In addition, we also establish sufficient conditions for the disease to go extinct with probability one in two cases. Meanwhile, we obtain a threshold parameter which can be utilized in identifying the stochastic extinction and persistence of the disease. One of the most important findings is that large environmental noises can suppress the outbreak of the disease. More precisely,

- If ${ {\mathcal R} }_{0}^{S}=\frac{{\sum }_{i=1}^{N}\,{\pi }_{i}{\beta }_{i}}{{\sum }_{i=1}^{N}\,{\pi }_{i}({\mu }_{i}+{\gamma }_{i}+{\varepsilon }_{i}+\frac{{\sigma }_{i}^{2}}{2})} > 1$, then there exists a unique ergodic stationary distribution *μ*(⋅,⋅) of system (1.4), which implies that the disease *I* is stochastic persistent in the mean a.s.
- If one of the following conditions holds

$$(i)\,{\hat{R}}^{\ast }=\frac{{\sum }_{i=1}^{N}\,{\pi }_{i}\frac{{\beta }_{i}^{2}}{2{\sigma }_{i}^{2}}}{{\sum }_{i=1}^{N}\,{\pi }_{i}({\mu }_{i}+{\gamma }_{i}+{\varepsilon }_{i})} < 1;$$

$$(ii)\,{\sigma }_{i}^{2}\le {\beta }_{i}\,{\rm{for}}\,{\rm{any}}\,i\in {\mathbb{S}}\,{\rm{and}}\,{ {\mathcal R} }_{0}^{S} < 1,$$

the disease *I* goes to extinction exponentially with probability one.

As is known to all, in order to obtain the ergodicity, we must prove that strong Feller property and irreducibility of Markov process. But in view of the proof of Lemma 3.2, model (1.4) is not irreducible. Therefore we use the Markov semigroup theory in^23,27^. Furthermore, in literatures^19–22,28^, the condition *γ*_*ij*_ > 0, *i* ≠ *j* is necessary, but in the present paper, this condition is not necessary and we only assume that *r*(*t*) is irreducible.

## Supplementary information

Appendix
