# Supplementary material for: A regime-switching SIR epidemic model with a ratio-dependent incidence rate and degenerate diffusion
Source: Sci Rep. 2019 Jul 23;9:10696. doi: 10.1038/s41598-019-47131-6 (PMC6650464; doi:10.1038/s41598-019-47131-6)
Supplement: Supplementary file 1 — Appendix [file 41598_2019_47131_MOESM1_ESM.pdf]

A regime-switching SIR epidemic model with a ratio-dependent incidence rate and degenerate diffusion

Zhongwei Cao[a], Xu Liu[a], Xiangdan Wen[b]*, Liya Liu[c], Li Zu[d]

a Department of Applied Mathematics, Jilin University of Finance and Economics,

Changchun 130117, Jilin Province, P.R. China

b Department of Mathematics, Yanbian University, Yanji 133002, Jilin Province, P. R. China

c College of Science, China University of Petroleum (East China),

Qingdao 266580, Shandong Province, P. R. China

d College of Mathematics and Statistics, Hainan Normal University, Haikou 571158, Hainan Province, P.R. China

*Correspondence and requests for materials should be addressed to X.L.(email:liuxu@jlufe.edu.cn) or X.W.(email:xdwen0502@yeah.net)

Appendix A. Preliminaries

In this section, some definitions and results about Markov semigroup and asymptotic properties [1, 2] are introduced to prove our main results.

1. Some useful lemmas

Detailed knowledge of Markov semigroup and Fokker-planck equation can be referred to reference [1, 2]. Here we just list two useful lemmas.

Lemma 1. Let $\{\mathcal{P}(t)\}_{t\geq 0}$ be a partially integral Markov semigroup. Assume that the semigroup $\{\mathcal{P}(t)\}_{t\geq 0}$ has only one invariant density f_*. If $f_* > 0$ a.e., then the semigroup $\{\mathcal{P}(t)\}_{t\geq 0}$ is asymptotically stable.

Lemma 2. Assume that a Markov semigroup $\{\mathcal{P}(t)\}_{t\geq 0}$ has the following properties:

(a) for every $f \in D$ we have $\int_0^\infty \mathcal{P}(t)f dt > 0$ a.e.

(b) for every $y_0 \in X$ there exist ϵ and a measurable function $\eta \geq 0$ such that $\int \eta(x)m(dx) > 0$ and

$$q(x,y) \geq \eta(x)\mathbf{1}_{B_\epsilon(y_0)}(y),$$

where q is a function satisfying $\int_X \int_X q(x,y)m(dx)m(dy) > 0$ and $\mathcal{P}(t_0)f(x) \geq \int_X q(x,y)f(y)m(dy)$ for every $f \in D$, $B_\epsilon(y_0)$ denotes an open ball of radius ϵ centered at y_0.

If the semigroup $\{\mathcal{P}(t)\}_{t\geq 0}$ has no invariant density then it is sweeping with respect to compact sets.

Remark 1. Here we give the precise meanings of the asymptotic behavior of a Markov semigroup. A density f_* is called invariant if $\mathcal{P}(t)f_* = f_*$ for each $t > 0$. The Markov semigroup $\{\mathcal{P}(t)\}_{t\geq 0}$ is called asymptotically stable if there is an invariant density f_* such that

$$\lim_{t\to\infty} \|\mathcal{P}(t)f - f_*\| = 0 \text{ for } f \in D.$$

A Markov semigroup $\{\mathcal{P}(t)\}_{t\geq 0}$ is called sweeping with respect to a set $A \in \Sigma$ if for every $f \in D$,

$$\lim_{t\to\infty} \int_A \mathcal{P}(t)f(x)m(dx) = 0.$$

2. Existence of global positive solutions to system (1.4)

Lemma 3. For any initial value $(S_0, I_0, r(0)) \in \mathbb{R}_+^2 \times \mathbb{S}$, there exists a unique solution $(S_t, I_t, r(t))$ of system (1.4) on $t \geq 0$ and the solution will remain in $\mathbb{R}_+^2 \times \mathbb{S}$ with probability one, namely, $(S_t, I_t, r(t)) \in \mathbb{R}_+^2 \times \mathbb{S}$ for all $t \geq 0$ almost surely (a.s.).

Proof. Since the coefficients of system (1.4) satisfy the local Lipschitz condition, then for any initial value $(S_0, I_0, r(0)) \in \mathbb{R}_+^2 \times \mathbb{S}$, there is a unique local solution $(S_t, I_t, r(t))$ on $t \in [0, \tau_e)$, where τ_e denotes the explosion time [3]. To prove this solution is global, we only need to verify $\tau_e = \infty$ a.s. To this end, let $n_0 \geq 1$ be sufficiently large such that S_0 and I_0 all lie within the interval $[\frac{1}{n_0}, n_0]$. For each integer $n \geq n_0$, define the stopping time

$$\tau_n = \inf\left\{t \in [0, \tau_e) : \min\{S_t, I_t\} \leq \frac{1}{n} \text{ or } \max\{S_t, I_t\} \geq n\right\},$$

where throughout this paper, we set $\inf \emptyset = \infty$ (as usual $\emptyset$ denotes the empty set). It is clear that τ_n is increasing as $n \to \infty$. Let $\tau_\infty = \lim_{n\to\infty} \tau_n$, whence $\tau_\infty \leq \tau_e$ a.s. If $\tau_\infty = \infty$ a.s. is true, then $\tau_e = \infty$ a.s. and $(S_t, I_t, r(t)) \in \mathbb{R}_+^2 \times \mathbb{S}$ a.s. for all $t \geq 0$. That is to say, to complete the proof, we only need to verify $\tau_\infty = \infty$ a.s. If this statement is not true, then there exists a pair of constants $T > 0$ and $\epsilon \in (0,1)$ such that

$$\mathbb{P}\{\tau_\infty \leq T\} > \epsilon.$$

Thus there is an integer $n_1 \geq n_0$ such that

$$\mathbb{P}\{\tau_n \leq T\} \geq \epsilon \text{ for any } n \geq n_1.$$

Define a C^2-function $\bar{V} : \mathbb{R}_+^2 \times \mathbb{S} \to \mathbb{R}_+ \bigcup\{0\}$ by

$$\bar{V}(S, I, r) = (S - 1 - \ln S) + (I - 1 - \ln I).$$

The nonnegativity of this function can be seen from

$$u - 1 - \ln u \geq 0 \text{ for any } u > 0.$$

Let $n \geq n_1$ and $T > 0$ be arbitrary. For any $0 \leq t \leq \tau_n \wedge T = \min\{\tau_n, T\}$, using the generalized Itô's formula [3] to $\bar{V}$ leads to

$$\begin{aligned}
d\bar{V}(S,I,r) &= \left(1-\frac{1}{S}\right)\left[\left(\Lambda_{r(t)} - \mu_{r(t)}S - \frac{\beta_{r(t)}S^h I}{S^h + \alpha_{r(t)}I^h}\right)dt - \frac{\sigma_{r(t)}S^h I}{S^h + \alpha_{r(t)}I^h}dB_t\right] + \frac{\sigma_{r(t)}^2 S^{2h-2}I^2}{2(S^h + \alpha_{r(t)}I^h)^2}dt \\
&\quad + \left(1-\frac{1}{I}\right)\left[\left(\frac{\beta_{r(t)}S^h I}{S^h + \alpha_{r(t)}I^h} - (\mu_{r(t)} + \gamma_{r(t)} + \varepsilon_{r(t)})I\right)dt + \frac{\sigma_{r(t)}S^h I}{S^h + \alpha_{r(t)}I^h}dB_t\right] \\
&\quad + \frac{\sigma_{r(t)}^2 S^{2h}}{2(S^h + \alpha_{r(t)}I^h)^2}dt \\
&= \left[\left(1-\frac{1}{S}\right)\left(\Lambda_{r(t)} - \mu_{r(t)}S - \frac{\beta_{r(t)}S^h I}{S^h + \alpha_{r(t)}I^h}\right) + \frac{\sigma_{r(t)}^2 S^{2h-2}I^2}{2(S^h + \alpha_{r(t)}I^h)^2}\right. \\
&\quad \left. + \left(1-\frac{1}{I}\right)\left(\frac{\beta_{r(t)}S^h I}{S^h + \alpha_{r(t)}I^h} - (\mu_{r(t)} + \gamma_{r(t)} + \varepsilon_{r(t)})I\right) + \frac{\sigma_{r(t)}^2 S^{2h}}{2(S^h + \alpha_{r(t)}I^h)^2}\right]dt \\
&\quad + \frac{\sigma_{r(t)}S^{h-1}I}{S^h + \alpha_{r(t)}I^h}dB_t - \frac{\sigma_{r(t)}S^h}{S^h + \alpha_{r(t)}I^h}dB_t,
\end{aligned}$$

where $L\bar{V} : \mathbb{R}_+^2 \times \mathbb{S} \to \mathbb{R}$ is defined by

$$\begin{aligned}
L\bar{V}(S,I,i) &= \left(1-\frac{1}{S}\right)\left(\Lambda_i - \mu_i S - \frac{\beta_i S^h I}{S^h + \alpha_i I^h}\right) + \frac{\sigma_i^2 S^{2h-2}I^2}{2(S^h + \alpha_i I^h)^2} + \left(1-\frac{1}{I}\right)\left(\frac{\beta_i S^h I}{S^h + \alpha_i I^h}\right. \\
&\quad \left. - (\mu_i + \gamma_i + \varepsilon_i)I\right) + \frac{\sigma_i^2 S^{2h}}{2(S^h + \alpha_i I^h)^2} \\
&= \Lambda_i + 2\mu_i + \gamma_i + \varepsilon_i + \frac{\beta_i S^{h-1}I}{S^h + \alpha_i I^h} + \frac{\sigma_i^2}{2}\left(\frac{S^{h-1}I}{S^h + \alpha_i I^h}\right)^2 + \frac{\sigma_i^2}{2}\left(\frac{S^h}{S^h + \alpha_i I^h}\right)^2 - \frac{\beta_i S^h}{S^h + \alpha_i I^h} \\
&\quad - \mu_i(S+I) - (\gamma_i + \varepsilon_i)I - \frac{\Lambda_i}{S} \\
&< \check{\Lambda} + 2\check{\mu} + \check{\gamma} + \check{\varepsilon} + \frac{\check{\sigma}^2}{2} + \frac{\check{\beta}(\frac{h-1}{h}S^h + \frac{1}{h}I^h)}{S^h + \hat{\alpha}I^h} + \frac{\check{\sigma}^2}{2}\left(\frac{\frac{h-1}{h}S^h + \frac{1}{h}I^h}{S^h + \hat{\alpha}I^h}\right)^2 \\
&\leq \check{\Lambda} + 2\check{\mu} + \check{\gamma} + \check{\varepsilon} + \frac{\check{\sigma}^2}{2} + \check{\beta}\left(\frac{h-1}{h} + \frac{1}{h\hat{\alpha}}\right) + \frac{\check{\sigma}^2}{2}\left(\frac{h-1}{h} + \frac{1}{h\hat{\alpha}}\right)^2 \\
&:= K_1,
\end{aligned}$$

where in the second inequality we have used the Young inequality $S^{h-1}I \leq \frac{h-1}{h}S^h + \frac{1}{h}I^h$ and K_1 is a positive constant independent of S, I and i. The rest of the proof is similar to Theorem 3.1 of Mao et al. [4] and so we omit it here. This completes the proof.

Remark 2. Since system (1.4) has a unique positive solution with probability one for any initial value $(S_0, I_0, r(0)) \in \mathbb{R}_+^2 \times \mathbb{S}$, then for any $i \in \mathbb{S}$,

$$\frac{d}{dt}(S_t + I_t) = \Lambda_i - \mu_i S_t - (\mu_i + \gamma_i + \varepsilon_i)I_t \text{ a.s.},$$

which implies that

$$\Lambda_i - (\mu_i + \gamma_i + \varepsilon_i)(S_t + I_t) < \frac{d}{dt}(S_t + I_t) < \Lambda_i - \mu_i(S_t + I_t) \text{ a.s.}$$

Therefore, the region

$$E := \left\{(x,y) \in \mathbb{R}_+^2 : \max_{i \in \mathbb{S}}\left\{\frac{\Lambda_i}{\mu_i + \gamma_i + \varepsilon_i}\right\} < x + y < \min_{i \in \mathbb{S}}\left\{\frac{\Lambda_i}{\mu_i}\right\}\right\} \tag{1}$$

is a positively invariant set of system (1.4). Thus from now on, we always assume that the initial value $(S_0, I_0, r(0)) \in E \times \mathbb{S}$.

Appendix B. Proof of Theorem 2.1

In order to verify Theorem 2.1, in view of the preliminaries in Appendix A, it only needs to show that the Markov semigroup formed by system (1.4) is asymptotic. We will prove this by Lemmas 4-8.

Lemma 4. The semigroup $\{\mathcal{T}_i(t)\}_{t\geq 0}$ is an integral Markov semigroup.

Proof. If $a(x)$ and $b(x)$ are vector fields on $\mathbb{R}^d$, then the Lie bracket $[a,b]$ is a vector field given by

$$[a,b]_j(x) = \sum_{k=1}^{d}\left(a_k\frac{\partial b_j}{\partial x_k}(x) - b_k\frac{\partial a_j}{\partial x_k}(x)\right),\ j=1,2,\ldots,d.$$

Let

$$a(x,y) = \begin{bmatrix} \Lambda_i - \mu_i x - \dfrac{\beta_i x^h y}{x^h+\alpha_i y^h} \\ \dfrac{\beta_i x^h y}{x^h+\alpha_i y^h} - (\mu_i+\gamma_i+\varepsilon_i)y \end{bmatrix},\quad b(x,y) = \begin{bmatrix} -\dfrac{\sigma_i x^h y}{x^h+\alpha_i y^h} \\ \dfrac{\sigma_i x^h y}{x^h+\alpha_i y^h} \end{bmatrix},$$

where $(x,y)\in E$.

Direct calculation leads to

$$\begin{aligned}[a,b] &= \begin{bmatrix} a_1\dfrac{\partial b_1}{\partial x} - b_1\dfrac{\partial a_1}{\partial x} + a_2\dfrac{\partial b_1}{\partial y} - b_2\dfrac{\partial a_1}{\partial y} \\ a_1\dfrac{\partial b_2}{\partial x} - b_1\dfrac{\partial a_2}{\partial x} + a_2\dfrac{\partial b_2}{\partial y} - b_2\dfrac{\partial a_2}{\partial y} \end{bmatrix} \\ &= \begin{bmatrix} \frac{h\alpha_i\mu_i\sigma_i x^h y^{h+1}}{(x^h+\alpha_i y^h)^2} - \frac{h\Lambda_i\alpha_i\sigma_i x^{h-1}y^{h+1}}{(x^h+\alpha_i y^h)^2} - \frac{\mu_i\sigma_i x^h y}{x^h+\alpha_i y^h} + \frac{\sigma_i(\mu_i+\gamma_i+\varepsilon_i)x^{2h}y}{(x^h+\alpha_i y^h)^2} + \frac{(1-h)\alpha_i\sigma_i(\mu_i+\gamma_i+\varepsilon_i)x^h y^{h+1}}{(x^h+\alpha_i y^h)^2} \\ \frac{h\Lambda_i\alpha_i\sigma_i x^{h-1}y^{h+1}}{(x^h+\alpha_i y^h)^2} - \frac{h\mu_i\alpha_i\sigma_i x^h y^{h+1}}{(x^h+\alpha_i y^h)^2} + \frac{\sigma_i(\mu_i+\gamma_i+\varepsilon_i)x^h y}{x^h+\alpha_i y^h} - \frac{\sigma_i(\mu_i+\gamma_i+\varepsilon_i)x^{2h}y}{(x^h+\alpha_i y^h)^2} - \frac{(1-h)\alpha_i\sigma_i(\mu_i+\gamma_i+\varepsilon_i)x^h y^{h+1}}{(x^h+\alpha_i y^h)^2} \end{bmatrix}.\end{aligned}$$

Noting that $b_1 = -b_2$, we have

$$\begin{aligned}\begin{vmatrix} [a,b] & b \end{vmatrix} &= \begin{vmatrix} a_1\dfrac{\partial b_1}{\partial x} - b_1\dfrac{\partial a_1}{\partial x} + a_2\dfrac{\partial b_1}{\partial y} - b_2\dfrac{\partial a_1}{\partial y} & b_1 \\ a_1\dfrac{\partial b_2}{\partial x} - b_1\dfrac{\partial a_2}{\partial x} + a_2\dfrac{\partial b_2}{\partial y} - b_2\dfrac{\partial a_2}{\partial y} & b_2 \end{vmatrix} \\ &= b_1^2\left(\frac{\partial a_1}{\partial x} + \frac{\partial a_2}{\partial x} - \frac{\partial a_1}{\partial y} - \frac{\partial a_2}{\partial y}\right) \\ &= \frac{(\gamma_i+\varepsilon_i)\sigma_i^2 x^{2h}y^2}{(x^h+\alpha_i y^h)^2} \\ &> 0,\end{aligned}$$

which means that b, $[a,b]$ are linearly independent on E.

Thus for every $(x,y)\in E$, vectors $b(x,y)$, $[a,b](x,y)$ span the space E. In view of Hörmander's Theorem [5], the transition probability function of $(S^{(i)}(t), I^{(i)}(t))$ has a smooth density $k_i \in C^\infty((0,\infty)\times E\times E)$. Then for every $f\in L^1(E,\mathcal{B}(E),m)$ satisfying $f\geq 0$ and $\|f\|=1$,

$$\mathcal{T}_i(t)f(x,y) = \iint_{\mathbb{R}_+^2} k_i(t,x,y;u,v)f(u,v)dudv.$$

Hence the semigroup $\{\mathcal{T}_i(t)\}_{t\geq 0}$ is an integral Markov semigroup. This completes the proof.

Lemma 5. For every $f\in D$,

$$\int_0^\infty \mathcal{T}_i(t)fdt > 0 \text{ a.e. on } E,$$

where D is given in (1).

Proof. For any $(x_0,y_0)\in E$, consider the following control system

$$\begin{cases} x_\phi'(t) = \Lambda_i - \mu_i x_\phi(t) - \dfrac{\beta_i x_\phi^h(t)y_\phi(t)}{x_\phi^h(t)+\alpha_i y_\phi^h(t)} - \dfrac{\sigma_i\phi x_\phi^h(t)y_\phi(t)}{x_\phi^h(t)+\alpha_i y_\phi^h(t)}, \\ y_\phi'(t) = \dfrac{\beta_i x_\phi^h(t)y_\phi(t)}{x_\phi^h(t)+\alpha_i y_\phi^h(t)} - (\mu_i+\gamma_i+\varepsilon_i)y_\phi(t) + \dfrac{\sigma_i\phi x_\phi^h(t)y_\phi(t)}{x_\phi^h(t)+\alpha_i y_\phi^h(t)}, \end{cases} \tag{2}$$

with the initial value $(x_\phi(0), y_\phi(0)) = (x_0, y_0)$.

Let $D_{x_0,y_0;\phi}$ be the Frechét derivative of the function $h \mapsto \mathbf{x}_{\phi+h}(T)$ from $L^2([0,T];\mathbb{R})$ to $\mathbb{R}^2$, where $\mathbf{x}_{\phi+h} = \begin{bmatrix} x_{\phi+h} \\ y_{\phi+h} \end{bmatrix}$. The derivative $D_{x_0,y_0;\phi}$ can be given by

$$D_{x_0,y_0;\phi}h = \int_0^T Q(T,s)\mathbf{g}h(s)ds,$$

where $\mathbf{g} = \begin{bmatrix} -\frac{\sigma_i x^h y}{x^h+\alpha_i y^h} \\ \frac{\sigma_i x^h y}{x^h+\alpha_i y^h} \end{bmatrix}$, $Q(t,t_0)$ $(T \geq t \geq t_0 \geq 0)$ is a matrix function such that $Q(t_0,t_0) = I$, $\frac{\partial Q(t,t_0)}{\partial t} = \digamma(t)Q(t,t_0)$ and $\digamma(t) = \mathbf{f}'(x_\phi(t), y_\phi(t)) + \mathbf{g}'(x_\phi(t), y_\phi(t))\phi$, where $\mathbf{f}'$ and $\mathbf{g}'$ are the Jacobians of

$$\mathbf{f} = \begin{bmatrix} \Lambda_i - \mu_i x - \dfrac{\beta_i x^h y}{x^h+\alpha_i y^h} \\ \dfrac{\beta_i x^h y}{x^h+\alpha_i y^h} - (\mu_i+\gamma_i+\varepsilon_i)y \end{bmatrix}$$

and

$$\mathbf{g} = \begin{bmatrix} -\dfrac{\sigma_i x^h y}{x^h+\alpha_i y^h} \\ \dfrac{\sigma_i x^h y}{x^h+\alpha_i y^h} \end{bmatrix},$$

respectively.

If the following two conditions hold:

1. The derivative $D_{x_0,y_0;\phi}$ has rank 2;

2. For any given two points $(x_0,y_0) \in E$ and $(x,y) \in E$ there is $T > 0$ and a smooth control function ϕ such that the solution of system (2) satisfies $x_\phi(0) = x_0$, $y_\phi(0) = y_0$, $x_\phi(T) = x$ and $y_\phi(T) = y$, where E is given in (1).

Then $k_i(T,x,y;x_0,y_0) > 0$ (see [6, 7, 8]). If this is the case, we obtain

$$\int_0^\infty \mathcal{T}_i(t)f dt > 0 \text{ a.e. on } E.$$

Thus the remaining of the proof is to check conditions 1 and 2.

First of all, we check the condition 1. Let $\epsilon \in (0,T)$ and $h(t) = \frac{\mathbf{1}_{[T-\epsilon,T]}(t)(x_\phi^h(t)+\alpha_i y_\phi^h(t))}{x_\phi^h(t)y_\phi(t)}$, $t \in [0,T]$, where $\mathbf{1}_{[T-\epsilon,T]}$ denotes the indicator function of the interval $[T-\epsilon,T]$. Since $Q(T,s) = I + \digamma(T)(T-s) + o(T-s)$, we get

$$D_{x_0,y_0;\phi}h = \epsilon\mathbf{v} + \frac{\epsilon^2}{2}\digamma(T)\mathbf{v} + o(\epsilon^2), \quad \mathbf{v} = \begin{bmatrix} -\sigma_i \\ \sigma_i \end{bmatrix},$$

$$\digamma(T)\mathbf{v} = \sigma_i \begin{bmatrix} b_1 \\ b_2 \end{bmatrix},$$

where $x = x_\phi(T)$, $y = y_\phi(T)$ and

$$\begin{aligned} b_1 &= \mu_i + \frac{h\alpha_i\beta_i x^{h-1}y^{h+1}}{(x^h+\alpha_i y^h)^2} + \frac{\phi h\alpha_i\sigma_i x^{h-1}y^{h+1}}{(x^h+\alpha_i y^h)^2} - \frac{\beta_i x^{2h}}{(x^h+\alpha_i y^h)^2} - \frac{(1-h)\alpha_i\beta_i x^h y^h}{(x^h+\alpha_i y^h)^2} \\ &\quad - \frac{\phi\sigma_i x^{2h}}{(x^h+\alpha_i y^h)^2} - \frac{\phi(1-h)\alpha_i\sigma_i x^h y^h}{(x^h+\alpha_i y^h)^2}, \end{aligned}$$

$$\begin{aligned} b_2 &= -\frac{h\alpha_i\beta_i x^{h-1}y^{h+1}}{(x^h+\alpha_i y^h)^2} - \frac{\phi h\alpha_i\sigma_i x^{h-1}y^{h+1}}{(x^h+\alpha_i y^h)^2} + \frac{\beta_i x^{2h}}{(x^h+\alpha_i y^h)^2} + \frac{(1-h)\alpha_i\beta_i x^h y^h}{(x^h+\alpha_i y^h)^2} \\ &\quad -(\mu_i+\gamma_i+\varepsilon_i) + \frac{\phi\sigma_i x^{2h}}{(x^h+\alpha_i y^h)^2} + \frac{\phi(1-h)\alpha_i\sigma_i x^h y^h}{(x^h+\alpha_i y^h)^2}. \end{aligned}$$

Hence $\mathbf{v}$ and $F(T)\mathbf{v}$ are linearly independent for any $(x,y)\in E$. This implies that $D_{x_0,y_0;\phi}$ has rank 2 for every $(x,y)\in E$.

Next, we prove the condition 2 holds. Let $z_\phi = x_\phi + y_\phi$, then system (2) becomes

$$\begin{cases} x'_\phi(t) = \Lambda_i - \mu_i x_\phi(t) - \dfrac{\beta_i x^h_\phi(t)(z_\phi(t)-x_\phi(t))}{x^h_\phi(t)+\alpha_i(z_\phi(t)-x_\phi(t))^h} - \dfrac{\sigma_i \phi x^h_\phi(t)(z_\phi(t)-x_\phi(t))}{x^h_\phi(t)+\alpha_i(z_\phi(t)-x_\phi(t))^h}, \\ z'_\phi(t) = \Lambda_i + (\gamma_i+\varepsilon_i)x_\phi(t) - (\mu_i+\gamma_i+\varepsilon_i)z_\phi(t). \end{cases} \tag{3}$$

Let

$$E_0 = \left\{(x,z)\in\mathbb{R}^2_+ : 0 < x < \min_{i\in\mathbb{S}}\left\{\frac{\Lambda_i}{\mu_i}\right\}, \max_{i\in\mathbb{S}}\left\{\frac{\Lambda_i}{\mu_i+\gamma_i+\varepsilon_i}\right\} < z < \min_{i\in\mathbb{S}}\left\{\frac{\Lambda_i}{\mu_i}\right\} \text{ and } x < z\right\}.$$

Now we claim that for any $(x_0,z_0)\in E_0$ and $(x_1,z_1)\in E_0$, there exists a control function ϕ and $T>0$ such that $x_\phi(0)=x_0$, $z_\phi(0)=z_0$, $x_\phi(T)=x_1$ and $z_\phi(T)=z_1$. If this is the case, we can obtain that for any two points $(x_0,y_0)\in E$ and $(x,y)\in E$ there is a control function ϕ and $T>0$ such that $x_\phi(0)=x_0$, $y_\phi(0)=y_0$, $x_\phi(T)=x$ and $y_\phi(T)=y$.

Next, we prove that our statement holds. Firstly, we find a positive constant T and a differentiable function

$$z_\phi : [0,T] \to \left(\max_{i\in\mathbb{S}}\left\{\frac{\Lambda_i}{\mu_i+\gamma_i+\varepsilon_i}\right\}, \min_{i\in\mathbb{S}}\left\{\frac{\Lambda_i}{\mu_i}\right\}\right)$$

such that $z_\phi(0)=z_0$, $z_\phi(T)=z_1$, $z'_\phi(0):=z_0^d$, $z'_\phi(T):=z_T^d$ and

$$\Lambda_i - (\mu_i+\gamma_i+\varepsilon_i)z_\phi(t) < z'_\phi(t) < \Lambda_i - \mu_i z_\phi(t),\ t\in[0,T]. \tag{4}$$

We split the construction of the function z_ϕ on three intervals $[0,\tau]$, $[\tau,T-\tau]$ and $[T-\tau,T]$, where $0<\tau<\frac{T}{2}$.

Let

$$\theta = \frac{1}{2}\min\left\{z_0 - \max_{i\in\mathbb{S}}\left\{\frac{\Lambda_i}{\mu_i+\gamma_i+\varepsilon_i}\right\}, z_1 - \max_{i\in\mathbb{S}}\left\{\frac{\Lambda_i}{\mu_i+\gamma_i+\varepsilon_i}\right\}, \min_{i\in\mathbb{S}}\left\{\frac{\Lambda_i}{\mu_i}\right\} - z_0, \min_{i\in\mathbb{S}}\left\{\frac{\Lambda_i}{\mu_i}\right\} - z_1\right\}.$$

When $z_\phi \in \left(\max_{i\in\mathbb{S}}\left\{\frac{\Lambda_i}{\mu_i+\gamma_i+\varepsilon_i}\right\}+\theta, \min_{i\in\mathbb{S}}\left\{\frac{\Lambda_i}{\mu_i}\right\}-\theta\right)$, we get

$$\Lambda_i - (\mu_i+\gamma_i+\varepsilon_i)z_\phi(t) < -(\mu_i+\gamma_i+\varepsilon_i)\theta < 0,\ \Lambda_i - \mu_i z_\phi(t) > \mu_i\theta > 0,\ t\in[0,T]. \tag{5}$$

According to (5) and $z_0 \in \left(\max_{i\in\mathbb{S}}\left\{\frac{\Lambda_i}{\mu_i+\gamma_i+\varepsilon_i}\right\}+\theta, \min_{i\in\mathbb{S}}\left\{\frac{\Lambda_i}{\mu_i}\right\}-\theta\right)$, we can define a C^2-function

$$z_\phi : [0,\tau] \to \left(\max_{i\in\mathbb{S}}\left\{\frac{\Lambda_i}{\mu_i+\gamma_i+\varepsilon_i}\right\}+\theta, \min_{i\in\mathbb{S}}\left\{\frac{\Lambda_i}{\mu_i}\right\}-\theta\right)$$

such that

$$z_\phi(0)=z_0,\ z'_\phi(0)=z_0^d,\ z'_\phi(\tau)=0$$

and z_ϕ satisfies (4) for $t\in[0,\tau]$. Analogously, we define a C^2-function

$$z_\phi : [T-\tau,T] \to \left(\max_{i\in\mathbb{S}}\left\{\frac{\Lambda_i}{\mu_i+\gamma_i+\varepsilon_i}\right\}+\theta, \min_{i\in\mathbb{S}}\left\{\frac{\Lambda_i}{\mu_i}\right\}-\theta\right)$$

such that

$$z_\phi(T)=z_1,\ z'_\phi(T)=z_T^d,\ z'_\phi(T-\tau)=0$$

and z_ϕ satisfies (4) for $t\in[T-\tau,T]$.

Taking T sufficiently large we can extend the function

$$z_\phi : [0,\tau]\cup[T-\tau,T] \to \left(\max_{i\in\mathbb{S}}\left\{\frac{\Lambda_i}{\mu_i+\gamma_i+\varepsilon_i}\right\}+\theta, \min_{i\in\mathbb{S}}\left\{\frac{\Lambda_i}{\mu_i}\right\}-\theta\right)$$

to a C^2-function z_ϕ defined on the whole interval $[0,T]$ such that

$$-(\mu_i+\gamma_i+\varepsilon_i)\theta \le z'_\phi(t) \le \mu_i\theta,\ t\in[\tau,T-\tau]$$

and thus the function z_ϕ satisfies (4) on $[0,T]$ by virtue of (5). Consequently, we can find a C^1-function x_ϕ which satisfies the second equation of (3) and finally we can determine a smooth control function ϕ from the first equation of (3), which means that the condition 2 holds. This completes the proof.

By Lemma 4, we obtain that the transition probability function for the diffusion process $(S^{(i)}(t), I^{(i)}(t))$ $(i \in \mathbb{S})$ is absolutely continuous with respect to the Lebesgue measure.

Let $(S_t, I_t, r(t))$ be the unique solution of system (1.4) with $(S_0, I_0, r(0)) \in E \times \mathbb{S}$, then $(S_t, I_t, r(t))$ constitutes a Markov process on $E \times \mathbb{S}$. In view of Lemma 5.5 in [9], for every $t > 0$ the distribution of the process $(S_t, I_t, r(t))$ is absolutely continuous and its density $u = (u_1, \dots, u_N)$ with $u_i := u(t, x, y, i))$ satisfies the following master equation

$$\frac{\partial u}{\partial t} = \Gamma^T u + \mathcal{A}u, \tag{6}$$

where $\mathcal{A}u = (\mathcal{A}_1 u_1, \dots, \mathcal{A}_N u_N)^T$. In view of Lemma 4, for any $i \in \mathbb{S}$ the operator $\mathcal{A}_i$ generates an integral semigroup $\{\mathcal{T}_i(t)\}_{t\geq 0}$ on the space $L^1(E, \mathcal{B}(E), m)$.

Let $X = E \times \mathbb{S}$, Σ be the σ-algebra of Borel subsets of X, and $\hat{m}$ be the product measure on (X, Σ) given by $\hat{m}(B \times i) = m(B)$ for each $B \in \mathcal{B}(E)$ and $i \in \mathbb{S}$. Apparently, $\mathcal{A}u$ generates a Markov semigroup $\{\mathcal{T}(t)\}_{t\geq 0}$ on the space $L^1(X, \Sigma, \hat{m})$ which is given by

$$\mathcal{T}(t)f = (\mathcal{T}_1(t)f(x, y, 1), \dots, \mathcal{T}_N(t)f(x, y, N))^T, \ f \in L^1(X, \Sigma, \hat{m}).$$

Let λ be a constant such that $\lambda > \max_{1\leq i\leq N}\{-\gamma_{ii}\}$ and $Q = \lambda^{-1}\Gamma' + I$, then (6) becomes

$$\frac{\partial u}{\partial t} = \lambda Q u - \lambda u + \mathcal{A}u. \tag{7}$$

It is easy to see that Q is also a Markov operator on $L^1(X, \Sigma, \hat{m})$.

According to the Philips perturbation theorem [10], (7) generates a Markov semigroup $\{\mathcal{P}(t)\}_{t\geq 0}$ on the space $L^1(X)$ with the initial condition $u(0, x, y, k) = f(x, y, k)$ given by

$$\mathcal{P}(t)f = e^{-\lambda t} \sum_{n=0}^{\infty} \lambda^n S^{(n)}(t)f, \tag{8}$$

where $S^{(0)}(t) = \mathcal{T}(t)$ and

$$S^{(n+1)}(t)f = \int_0^t S^{(0)}(t-s) Q S^{(n)}(s) f ds, \ n \geq 0. \tag{9}$$

The semigroup $\{\mathcal{P}(t)\}_{t\geq 0}$ satisfies the following integral equation

$$\mathcal{P}(t)f = e^{-\lambda t}\mathcal{T}(t)f + \lambda \int_0^t e^{-\lambda s}\mathcal{T}(s) Q \mathcal{P}(t-s) f ds. \tag{10}$$

Lemma 6. For every $f \in D$,

$$\int_0^\infty \mathcal{P}(t) f dt > 0 \text{ a.e. on } X,$$

where D is given in (1).

Proof. Let

$$\Omega_i := \left\{ \mathbf{x} \in E : \int_0^\infty \mathcal{P}(t) f(\mathbf{x}, i) dt = 0 \right\}, \ i \in \mathbb{S}.$$

In order to prove this lemma it only needs to verify that $m(\Omega_i) = 0$, $i \in \mathbb{S}$. Since $\mathcal{P}(t)f(\mathbf{x}, i)$ is continuous on $(0, \infty)$ with respect to t, then for any $\mathbf{x} \in \Omega_i$, we have $\mathcal{P}(t)f(\mathbf{x}, i) = 0$, $t \in (0, \infty)$. (Here $\mathcal{P}(t)f(\mathbf{x}, i)$ should be understood as $(\mathcal{P}(t)f)(\mathbf{x}, i)$, and in the following the formulas concerning operators have the same meaning.) It follows from (8) that

$$S^{(n)}(t) f(\mathbf{x}, i) = 0, \ \mathbf{x} \in \Omega_i, \ n = 0, 1, \dots, \ t \in (0, \infty).$$

In view of (9) and the continuity of $S(t)$, we get that

$$S^{(0)}(t-s) Q S^{(n)}(s) f(\mathbf{x}, i) = 0, \ s \in [0, t], \ n = 0, 1, \dots.$$

Especially, for any $\mathbf{x} \in \Omega_i$, we obtain that $QS^{(n)}(t)f(\mathbf{x},i) = 0$ on $(0,\infty)$, which together with (9) implies

$$Q^n S^{(0)}(t)f(\mathbf{x},i) = Q^n \mathcal{T}(t)f(\mathbf{x},i) = 0,\ n = 1,2,\dots,\ t \in (0,\infty).$$

Hence $\Omega_i \subset \Omega_i'$, where

$$\Omega_i' := \left\{ \mathbf{x} \in E : \int_0^\infty Q^n \mathcal{T}(t)f(\mathbf{x},i)dt = 0,\ n = 1,2,\dots \right\}.$$

According to Lemma 5, we get that for any $i \in \mathbb{S}$,

$$\int_0^\infty \mathcal{T}_i(t)f(\mathbf{x},i)dt > 0 \text{ a.e. on } E.$$

It is clear that

$$\int_0^\infty Q\mathcal{T}(t)f(\mathbf{x},i)dt \geq (\lambda^{-1}\gamma_{ii}+1)\int_0^\infty \mathcal{T}_i(t)f(\mathbf{x},i)dt > 0 \text{ a.e. on } E. \tag{11}$$

Since the matrix Γ is irreducible, then for any state $j \in \mathbb{S}$ $(j \neq i)$ there exists some integer k such that $(Q^k)_{ij} > 0$ and

$$\int_0^\infty Q^k\mathcal{T}(t)f(\mathbf{x},i)dt \geq (Q^k)_{ij}\int_0^\infty \mathcal{T}_j(t)f(\mathbf{x},j)dt > 0 \text{ a.e. on } E. \tag{12}$$

In view of (11) and (12), we have $m(\Omega_i') = m(\Omega_i) = 0$ for any $i \in \mathbb{S}$. This completes the proof.

Making use of Lemmas 1 and 2 we can present the following result. The proof is similar to Corollary 1 in [11].

Lemma 7. The semigroup $\{\mathcal{P}(t)\}_{t\geq 0}$ is asymptotically stable or is sweeping with respect to compact sets.

Proof. Since $\{\mathcal{T}_i(t)\}_{t\geq 0}$ $(i \in \mathbb{S})$ is an integral Markov semigroup with a continuous kernel $k_i(t,x,y;u,v)$ for $t > 0$, then in view of (8), we can get that the semigroup $\{\mathcal{P}(t)\}_{t\geq 0}$ is partially integral.

If the semigroup $\{\mathcal{P}(t)\}_{t\geq 0}$ has an invariant density f_*, then by Lemma 6, we can obtain that $f_* > 0$ a.e. If a Markov semigroup has two different invariant densities then it has two invariant densities with disjoint supports, which is impossible in our case. Thus the semigroup $\{\mathcal{P}(t)\}_{t\geq 0}$ has at most one invariant density. Fixing $t > 0$ and $(u_0,v_0) \in E$. Since

$$\iint_E k_i(t,x,y;u_0,v_0)dxdy = 1,$$

there exists an $(x_0,y_0) \in E$ and a $\lambda > 0$ such that

$$k_i(t,x_0,y_0;u_0,v_0) > \lambda.$$

According to the continuity of the kernel k_i, we get that there exists an $\epsilon > 0$ such that $k_i(t,x,y;u,v) > \lambda$ for $(x,y) \in B_\epsilon(x_0,y_0)$ and $(u,v) \in B_\epsilon(u_0,v_0)$. Let $\eta(x,y) = \lambda \mathbf{1}_{B_\epsilon(x_0,y_0)}(x,y)$. Then

$$k_i(t,x,y;u,v) \geq \eta(x,y)$$

for any $(x,y) \in E$ and $(u,v) \in B_\epsilon(u_0,v_0)$. In view of (10), we can obtain that the condition (b) of Lemma 2 holds. Therefore, by Lemmas 1 and 2, we can immediately obtain the required statement. This completes the proof.

Lemma 8. If $\mathcal{R}_0^S = \frac{\sum_{i=1}^N \pi_i\beta_i}{\sum_{i=1}^N \pi_i(\mu_i+\gamma_i+\varepsilon_i+\frac{\sigma_i^2}{2})} > 1$, then the semigroup $\{\mathcal{P}(t)\}_{t\geq 0}$ is asymptotically stable.

Proof. In order to prove this lemma, we need to construct a nonnegative C^2-function V and a closed set $\mathcal{D} \in \mathcal{B}(E)$ (which lies entirely in E) such that for any $i \in \mathbb{S}$,

$$\sup_{(x,y)\in E\setminus\mathcal{D}} \mathscr{A}^*V(x,y,i) < 0,$$

where

$$\mathscr{A}^*V(x,y,i) = \frac{\sigma_i^2x^{2h}y^2}{2(x^h+\alpha_iy^h)^2}\left[\frac{\partial^2V}{\partial x^2} - 2\frac{\partial^2V}{\partial x\partial y} + \frac{\partial^2V}{\partial y^2}\right] + g_i^1\frac{\partial V}{\partial x} + g_i^2\frac{\partial V}{\partial y} + \sum_{j\neq i,j\in\mathbb{S}}\gamma_{ij}(V(x,y,j)-V(x,y,i))$$

and $g_i^1(x,y,i) = \Lambda_i - \mu_i x - \frac{\beta_i x^h y}{x^h+\alpha_i y^h}$, $g_i^2(x,y,i) = \frac{\beta_i x^h y}{x^h+\alpha_i y^h} - (\mu_i+\gamma_i+\varepsilon_i)y$.

We define a vector $R = (R_1, R_2, \dots, R_N)$ with $R_i = -\beta_i + \mu_i + \gamma_i + \varepsilon_i + \frac{\sigma_i^2}{2}$, $i = 1, 2, \dots, N$. Since the generator matrix Γ is irreducible, then for any R_i, there is $\omega = (\omega_1, \omega_2, \dots, \omega_N)^T$ which is a solution of the Poisson system (see [12], Lemma 2.3) such that

$$\Gamma\omega + R = \sum_{i=1}^{N} \pi_i R_i \mathbf{1},$$

where $\mathbf{1} = (1, \dots, 1)^T$. This implies that for any $i \in \mathbb{S}$,

$$\sum_{l \neq i \in \mathbb{S}} \gamma_{il}(\omega_l - \omega_i) - R_i = -\sum_{i\in\mathbb{S}} \pi_i R_i = -\mathcal{R}_0^s = -\sum_{i\in\mathbb{S}} \pi_i \Big(\mu_i + \gamma_i + \varepsilon_i + \frac{\sigma_i^2}{2}\Big)(\mathcal{R}_0^S - 1). \tag{13}$$

Taking a positive constant M sufficiently large such that

$$-M\sum_{i\in\mathbb{S}} \pi_i \Big(\mu_i + \gamma_i + \varepsilon_i + \frac{\sigma_i^2}{2}\Big)(\mathcal{R}_0^S - 1) + \Big(\check{\beta} + \frac{\check{\sigma}^2}{2}\Big)\Big(\frac{h-1}{h} + \frac{1}{h\hat{\alpha}}\Big) + 3\check{\mu} + \check{\gamma} + \check{\varepsilon} \le -2. \tag{14}$$

Define a C^2-function

$$Q(x,y,i) = -M\ln y - \ln x - \ln(\mathcal{L} - x - y) - \ln(x + y - \mathscr{L}),$$

for any $(x,y,i) \in E\times\mathbb{S}$, where $\mathcal{L} = \min_{i\in\mathbb{S}}\{\frac{\Lambda_i}{\mu_i}\}$, $\mathscr{L} = \max_{i\in\mathbb{S}}\{\frac{\Lambda_i}{\mu_i+\gamma_i+\varepsilon_i}\}$. It is not difficult to check that there is a unique point $(x_0(i), y_0(i), i)$ such that $Q(x_0(i), y_0(i), i)$ is the minimum value of the function $Q(x,y,i)$. Hence $Q(x,y,i) - Q(x_0(i), y_0(i), i) \ge 0$.

Next, we define a C^2-function $V : E \times \mathbb{S} \to \mathbb{R}_+$ by

$$\begin{aligned} V(x,y,i) &= Q(x,y,i) - Q(x_0(i), y_0(i), i) + M(\omega_i + |\omega|) \\ &= -M\ln y + M(\omega_i + |\omega|) - \ln x - \ln(\mathcal{L} - x - y) - \ln(x + y - \mathscr{L}) - Q(x_0(i), y_0(i), i), \end{aligned}$$

where $\omega = (\omega_1, \omega_2, \dots, \omega_N)^T$, $|\omega| = \sqrt{\omega_1^2 + \omega_2^2 + \ldots + \omega_N^2}$ and ω_i $(i \in \mathbb{S})$ will be determined later. $|\omega|$ here is used to make sure that $\omega_i + |\omega|$ is nonnegative. Such a function V is called a Khasminskiĭ function [13]. Denote by $V_1 = -\ln y$, $V_2 = \omega_i + |\omega|$, $V_3 = -\ln x$, $V_4 = -\ln(\mathcal{L} - x - y)$, $V_5 = -\ln(x + y - \mathscr{L}) - Q(x_0(i), y_0(i), i)$, Applying the generalized Itô's formula to V_1, we get

$$\begin{aligned} \mathscr{A}^* V_1 &= -\frac{\beta_i x^h}{x^h + \alpha_i y^h} + \mu_i + \gamma_i + \varepsilon_i + \frac{\sigma_i^2 x^{2h}}{2(x^h + \alpha_i y^h)^2} \le -\beta_i + \mu_i + \gamma_i + \varepsilon_i + \frac{\sigma_i^2}{2} + \frac{\check{\beta}\alpha_i y^h}{x^h + \alpha_i y^h} \\ &:= -R_i + \frac{\check{\beta}\alpha_i y^h}{x^h + \alpha_i y^h} \end{aligned}$$

and

$$\mathscr{A}^* V_2 = \sum_{l\neq k\in\mathbb{S}} \gamma_{kl}(\omega_l - \omega_k).$$

Thus

$$\begin{aligned} \mathscr{A}^*(V_1 + V_2) &\le M\Big[-R_i + \frac{\check{\beta}\alpha_i y^h}{x^h + \alpha_i y^h} + \sum_{l\neq k\in\mathbb{S}} \gamma_{kl}(\omega_l - \omega_k)\Big] \\ &= -M\sum_{i\in\mathbb{S}} \pi_i\Big(\mu_i + \gamma_i + \varepsilon_i + \frac{\sigma_i^2}{2}\Big)(\mathcal{R}_0^S - 1) + \frac{M\check{\beta}\alpha_i y^h}{x^h + \alpha_i y^h}, \end{aligned}$$

where in the above equality, we have used (13).

Similarly

$$\mathscr{A}^* V_3 = -\frac{\Lambda_i}{x} + \mu_i + \frac{\beta_i x^{h-1} y}{x^h + \alpha_i y^h} + \frac{\sigma_i^2 x^{2h-2} y^2}{2(x^h + \alpha_i y^h)^2} \le -\frac{\hat{\Lambda}}{x} + \check{\mu} + \frac{\check{\beta} x^{h-1} y}{x^h + \hat{\alpha} y^h} + \frac{\check{\sigma}^2 x^{2h-2} y^2}{2(x^h + \hat{\alpha} y^h)^2}$$

$$\leq \quad -\frac{\hat{\Lambda}}{x}+\breve{\mu}+\Big(\breve{\beta}+\frac{\breve{\sigma}^2}{2}\Big)\Big(\frac{h-1}{h}+\frac{1}{h\hat{\alpha}}\Big),$$

where in the second inequality we have used the Young inequality $x^{h-1}y \leq \frac{h-1}{h}x^h + \frac{1}{h}y^h$.

$$\mathscr{A}^*V_4 = \frac{1}{\mathcal{L}-x-y}(\Lambda_i - \mu_i(x+y) - (\gamma_i+\varepsilon_i)y) \leq \mu_i - \frac{(\gamma_i+\varepsilon_i)y}{\mathcal{L}-x-y} \leq \breve{\mu} - \frac{\hat{\gamma}y}{\mathcal{L}-x-y}$$

and

$$\begin{aligned}\mathscr{A}^*V_5 \quad &= \quad -\frac{1}{x+y-\mathscr{L}}(\Lambda_i+(\gamma_i+\varepsilon_i)x-(\mu_i+\gamma_i+\varepsilon_i)(x+y)) \leq \mu_i+\gamma_i+\varepsilon_i - \frac{(\gamma_i+\varepsilon_i)x}{x+y-\mathscr{L}} \\ &\leq \breve{\mu}+\breve{\gamma}+\breve{\varepsilon} - \frac{\hat{\gamma}x}{x+y-\mathscr{L}}.\end{aligned}$$

Therefore

$$\begin{aligned}\mathscr{A}^*V \quad \leq \quad & -M\sum_{i\in\mathbb{S}}\pi_i\Big(\mu_i+\gamma_i+\varepsilon_i+\frac{\sigma_i^2}{2}\Big)(\mathcal{R}_0^S-1) + \frac{M\breve{\beta}\alpha_i y^h}{x^h+\alpha_i y^h} - \frac{\hat{\Lambda}}{x} - \frac{\hat{\gamma}y}{\mathcal{L}-x-y} - \frac{\hat{\gamma}x}{x+y-\mathscr{L}} \\ & +\Big(\breve{\beta}+\frac{\breve{\sigma}^2}{2}\Big)\Big(\frac{h-1}{h}+\frac{1}{h\hat{\alpha}}\Big)+3\breve{\mu}+\breve{\gamma}+\breve{\varepsilon}.\end{aligned}$$

Define a bounded closed set as follows

$$\mathcal{D}_\epsilon = \{(x,y)\in E : \epsilon \leq x \leq \mathcal{L}, \epsilon^2 \leq y \leq \mathcal{L}, \mathscr{L}+\epsilon^2 \leq x+y \leq \mathcal{L}-\epsilon^3\},$$

where $0<\epsilon<1$ is a sufficiently small number. In the set $E\setminus\mathcal{D}_\epsilon$, we can choose ϵ sufficiently small such that the following conditions hold

$$-\frac{\hat{\Lambda}}{\epsilon}+K_2 < -1, \tag{15}$$

$$\epsilon < \frac{1}{\sqrt[h]{M\breve{\beta}\breve{\alpha}}}, \tag{16}$$

$$-\frac{\hat{\gamma}}{\epsilon}+K_2 < -1, \tag{17}$$

where K_2 is a positive constant which will be given explicitly in expression (18). For convenience, we can divide $E\setminus\mathcal{D}_\epsilon$ into the following four domains,

$$\mathcal{D}_\epsilon^1 = \{(x,y)\in E : x<\epsilon\},\ \mathcal{D}_\epsilon^2 = \{(x,y)\in E : \epsilon\leq x, y<\epsilon^2\},$$

$$\mathcal{D}_\epsilon^3 = \{(x,y)\in E : \epsilon^2 \leq y < \mathcal{L}, \mathcal{L}-\epsilon^3 < x+y < \mathcal{L}\},\ \mathcal{D}_\epsilon^4 = \{(x,y)\in E : \epsilon\leq x<\mathcal{L}, \mathscr{L}<x+y<\mathscr{L}+\epsilon^2\}.$$

Then $E\setminus\mathcal{D}_\epsilon = \mathcal{D}_\epsilon^1\bigcup\mathcal{D}_\epsilon^2\bigcup\mathcal{D}_\epsilon^3\bigcup\mathcal{D}_\epsilon^4$. Next, we will prove that $\mathscr{A}^*V(x,y,i) < -1$ on $(E\setminus\mathcal{D}_\epsilon)\times\mathbb{S}$, which is equivalent to proving it on the above four domains, respectively.

Case 1. For any $(x,y,i)\in\mathcal{D}_\epsilon^1\times\mathbb{S}$, we have

$$\mathscr{A}^*V \leq -\frac{\hat{\Lambda}}{x}+\frac{M\breve{\beta}\breve{\alpha}y^h}{x^h+\hat{\alpha}y^h}+\Big(\breve{\beta}+\frac{\breve{\sigma}^2}{2}\Big)\Big(\frac{h-1}{h}+\frac{1}{h\hat{\alpha}}\Big)+3\breve{\mu}+\breve{\gamma}+\breve{\varepsilon} \leq -\frac{\hat{\Lambda}}{x}+K_2 < -\frac{\hat{\Lambda}}{\epsilon}+K_2 < -1,$$

which follows from (15) and

$$K_2 = \sup_{(x,y)\in E}\Big\{\frac{M\breve{\beta}\breve{\alpha}y^h}{x^h+\hat{\alpha}y^h}+\Big(\breve{\beta}+\frac{\breve{\sigma}^2}{2}\Big)\Big(\frac{h-1}{h}+\frac{1}{h\hat{\alpha}}\Big)+3\breve{\mu}+\breve{\gamma}+\breve{\varepsilon}\Big\}. \tag{18}$$

Thus we can derive that for a sufficiently small ϵ,

$$\mathscr{A}^*V < -1 \text{ for any } (x,y,i)\in\mathcal{D}_\epsilon^1\times\mathbb{S}.$$

Case 2. On $\mathcal{D}_\epsilon^2 \times \mathbb{S}$, we obtain

$$\begin{aligned}
\mathscr{A}^*V &\leq -M\sum_{i\in\mathbb{S}}\pi_i\Big(\mu_i+\gamma_i+\varepsilon_i+\frac{\sigma_i^2}{2}\Big)(\mathcal{R}_0^S-1)+M\check{\beta}\check{\alpha}\Big(\frac{y}{x}\Big)^h \\
&\quad +\Big(\check{\beta}+\frac{\check{\sigma}^2}{2}\Big)\Big(\frac{h-1}{h}+\frac{1}{h\hat{\alpha}}\Big)+3\check{\mu}+\check{\gamma}+\check{\varepsilon} \\
&\leq -M\sum_{i\in\mathbb{S}}\pi_i\Big(\mu_i+\gamma_i+\varepsilon_i+\frac{\sigma_i^2}{2}\Big)(\mathcal{R}_0^S-1)+M\check{\beta}\check{\alpha}\epsilon^h \\
&\quad +\Big(\check{\beta}+\frac{\check{\sigma}^2}{2}\Big)\Big(\frac{h-1}{h}+\frac{1}{h\hat{\alpha}}\Big)+3\check{\mu}+\check{\gamma}+\check{\varepsilon} \\
&< -2+1 \\
&= -1,
\end{aligned}$$

which follows from (14) and (16) Hence we can get that for a sufficiently small ϵ,

$$\mathscr{A}^*V < -1 \text{ on } \mathcal{D}_\epsilon^2 \times \mathbb{S}.$$

Case 3. For any $(x,y,i) \in \mathcal{D}_\epsilon^3 \times \mathbb{S}$, we get

$$\begin{aligned}
\mathscr{A}^*V &\leq -\frac{\hat{\gamma}y}{\mathcal{L}-x-y}+\frac{M\check{\beta}\check{\alpha}y^h}{x^h+\hat{\alpha}y^h}+\Big(\check{\beta}+\frac{\check{\sigma}^2}{2}\Big)\Big(\frac{h-1}{h}+\frac{1}{h\hat{\alpha}}\Big)+3\check{\mu}+\check{\gamma}+\check{\varepsilon} \leq -\frac{\hat{\gamma}y}{\mathcal{L}-x-y}+K_2 \\
&< -\frac{\hat{\gamma}}{\epsilon}+K_2 < -1,
\end{aligned}$$

which follows from (17). Therefore, we can obtain that for a sufficiently small ϵ,

$$\mathscr{A}^*V < -1 \text{ for any } (x,y,i) \in \mathcal{D}_\epsilon^3 \times \mathbb{S}.$$

Case 4. If $(x,y,i) \in \mathcal{D}_\epsilon^4 \times \mathbb{S}$, we derive

$$\begin{aligned}
\mathscr{A}^*V &\leq -\frac{\hat{\gamma}x}{x+y-\mathscr{L}}+\frac{M\check{\beta}\check{\alpha}y^h}{x^h+\hat{\alpha}y^h}+\Big(\check{\beta}+\frac{\check{\sigma}^2}{2}\Big)\Big(\frac{h-1}{h}+\frac{1}{h\hat{\alpha}}\Big)+3\check{\mu}+\check{\gamma}+\check{\varepsilon} \leq -\frac{\hat{\gamma}x}{x+y-\mathscr{L}}+K_2 \\
&< -\frac{\hat{\gamma}}{\epsilon}+K_2 < -1,
\end{aligned}$$

which follows from (17). Consequently

$$\mathscr{A}^*V < -1 \text{ for any } (x,y,i) \in \mathcal{D}_\epsilon^4 \times \mathbb{S}.$$

In summary, for any $i \in \mathbb{S}$,

$$\sup_{(x,y,i)\in(E\setminus\mathcal{D}_\epsilon)\times\mathbb{S}} \mathscr{A}^*V(x,y,i) < -1.$$

Discussed in a similar way in [13], the existence of a Khasminskiǐ function means that the semigroup is not cleared from the set $\mathcal{D}_\epsilon$. According to Lemma 7, we can draw a conclusion that the semigroup $\{\mathcal{P}(t)\}_{t\geq 0}$ is asymptotically stable. This completes the proof.

Appendix C. Proof of Theorem 2.2

Proof. Using the generalized Itô's formula to $\ln I$, it is easy to see that

$$d\ln I = \left[\frac{\beta_{r(t)}S^h}{S^h+\alpha_{r(t)}I^h}-(\mu_{r(t)}+\gamma_{r(t)}+\varepsilon_{r(t)})-\frac{\sigma_{r(t)}^2}{2}\Big(\frac{S^h}{S^h+\alpha_{r(t)}I^h}\Big)^2\right]dt+\frac{\sigma_{r(t)}S^h}{S^h+\alpha_{r(t)}I^h}dB_t. \qquad (19)$$

Let $\frac{S^h}{S^h+\alpha_i I^h}=z$ and $z\in(0,1]$, we have

$$f(z) := \Big(\beta_i z-(\mu_i+\gamma_i+\varepsilon_i)-\frac{\sigma_i^2}{2}z^2\Big) = -\Big(\frac{\sigma_i}{\sqrt{2}}z-\frac{\sqrt{2}\beta_i}{2\sigma_i}\Big)^2+\frac{\beta_i^2}{2\sigma_i^2}-(\mu_i+\gamma_i+\varepsilon_i),\ i\in\mathbb{S}. \qquad (20)$$

Case 1. In view of (20), we have

$$f(z) \le \frac{\beta_i^2}{2\sigma_i^2} - (\mu_i + \gamma_i + \varepsilon_i),\ i \in \mathbb{S}.$$

Therefore we know from (19) that

$$d\ln I \le \frac{\beta_{r(t)}^2}{2\sigma_{r(t)}^2}dt - (\mu_{r(t)} + \gamma_{r(t)} + \varepsilon_{r(t)})dt + \frac{\sigma_{r(t)}S^h}{S^h + \alpha_{r(t)}I^h}dB_t := R_1^*(r(t))dt + \frac{\sigma_{r(t)}S^h}{S^h + \alpha_{r(t)}I^h}dB_t. \tag{21}$$

Integrating from 0 to t and then dividing by t on both sides of (21), we can conclude that

$$\frac{\ln I_t - \ln I_0}{t} \le \frac{1}{t}\int_0^t R_1^*(r(s))ds + \frac{1}{t}\int_0^t \frac{\sigma_{r(s)}S_s^h}{S_s^h + \alpha_{r(s)}I_s^h}dB_s = \frac{1}{t}\int_0^t R_1^*(r(s))ds + \frac{M_t}{t}, \tag{22}$$

where $M_t := \int_0^t \frac{\sigma_{r(s)}S_s^h}{S_s^h + \alpha_{r(s)}I_s^h}dB_s$ is a local martingale whose quadratic variation is

$$\langle M, M\rangle_t = \int_0^t \frac{\sigma_{r(s)}^2 S_s^{2h}}{(S_s^h + \alpha_{r(s)}I_s^h)^2}ds.$$

Then we get

$$\limsup_{t\to\infty}\frac{\langle M, M\rangle_t}{t} = \limsup_{t\to\infty}\frac{1}{t}\int_0^t \frac{\sigma_{r(s)}^2 S_s^{2h}}{(S_s^h + \alpha_{r(s)}I_s^h)^2}ds \le \check{\sigma}^2 \limsup_{t\to\infty}\frac{1}{t}\int_0^t 1ds = \check{\sigma}^2 < \infty \text{ a.s.}$$

Using the Strong Law of Large Numbers for local martingale [14] leads to

$$\lim_{t\to\infty}\frac{M_t}{t} = 0 \text{ a.s.} \tag{23}$$

By the ergodic property of $r(t)$, we get

$$\lim_{t\to\infty}\frac{1}{t}\int_0^t R_1^*(r(s))ds = \sum_{i=1}^N \pi_i R_1^*(i). \tag{24}$$

Taking the superior limit on the both sides of (22) and combining with (23) and (24), we obtain

$$\limsup_{t\to\infty}\frac{\ln I(t)}{t} \le \sum_{i=1}^N \pi_i R_1^*(i) = \sum_{i=1}^N \pi_i(\mu_i + \gamma_i + \varepsilon_i)(\bar{R}^* - 1) < 0 \text{ a.s.},$$

where

$$\bar{R}^* = \frac{\sum_{i=1}^N \pi_i \frac{\beta_i^2}{2\sigma_i^2}}{\sum_{i=1}^N \pi_i(\mu_i + \gamma_i + \varepsilon_i)}.$$

Case 2. When $\frac{\sigma_i}{\sqrt{2}} \le \frac{\sqrt{2}\beta_i}{2\sigma_i}$, that is $\sigma_i^2 \le \beta_i$, $i \in \mathbb{S}$, then $f(z) \le f(1)$, Thus

$$f(z) \le \beta_i - \left(\mu_i + \gamma_i + \varepsilon_i + \frac{\sigma_i^2}{2}\right),\ i \in \mathbb{S}.$$

Hence

$$d\ln I \le \beta_{r(t)}dt - \left(\mu_{r(t)} + \gamma_{r(t)} + \varepsilon_{r(t)} + \frac{\sigma_{r(t)}^2}{2}\right)dt + \frac{\sigma_{r(t)}S^h}{S^h + \alpha_{r(t)}I^h}dB_t := R_2^*(r(t))dt + \frac{\sigma_{r(t)}S^h}{S^h + \alpha_{r(t)}I^h}dB_t. \tag{25}$$

Integrating from 0 to t and then dividing by t on both sides of (25) yield

$$\frac{\ln I_t - \ln I_0}{t} \le \frac{1}{t}\int_0^t R_2^*(r(s))ds + \frac{1}{t}\int_0^t \frac{\sigma_{r(s)}S_s^h}{S_s^h + \alpha_{r(s)}I_s^h}dB_s = \frac{1}{t}\int_0^t R_2^*(r(s))ds + \frac{M_t}{t}. \tag{26}$$

According to the ergodic property of $r(t)$, we have

$$\lim_{t\to\infty}\frac{1}{t}\int_0^t R_2^*(r(s))ds = \sum_{i=1}^{N}\pi_i R_2^*(i). \tag{27}$$

Taking the superior limit on the both sides of (26) and making using of (23) and (27), we obtain

$$\limsup_{t\to\infty}\frac{\ln I(t)}{t} \le \sum_{i=1}^{N}\pi_i R_2^*(i) = \sum_{i=1}^{N}\pi_i\left(\mu_i+\gamma_i+\varepsilon_i+\frac{\sigma_i^2}{2}\right)(\mathcal{R}_0^S-1) < 0 \text{ a.s.},$$

where

$$\mathcal{R}_0^S = \frac{\sum_{i=1}^N \pi_i\beta_i}{\sum_{i=1}^N \pi_i(\mu_i+\gamma_i+\varepsilon_i+\frac{\sigma_i^2}{2})},$$

which implies that $\lim_{t\to\infty} I(t) = 0$ a.s. In other words, the disease I tends to zero exponentially with probability one. This completes the proof.

References

[1] Rudnicki, R. On asymptotic stability and sweeping for Markov operators. Bull. Pol. Acad. Sci. Math. **43**, 245-262 (1995).

[2] Pichór, K. & Rudnicki, R. Continuous Markov Semigroups and Stability of Transport Equations. J. Math. Anal. Appl. **249**, 668-685 (2000).

[3] Mao, X. & Yuan, C. Stochastic Differential Equations With Markovian Switching (Imperial College Press, London, 2006).

[4] Mao, X., Marion, G. & Renshaw, E. Environmental noise suppresses explosion in population dynamics. Stoch. Process. Appl. **97**, 95-110 (2002).

[5] Bell, D.R. The Malliavin Calculus (Dover publications, New York, 2006).

[6] Aida, S., Kusuoka, S. & Strook, D. On the support of Wiener functionals in *Asymptotic Problems in Probability Theory: Wiener Functionals and Asymptotic* (eds. Elworthy, K.D. & Ikeda, N.) 3-34 (Longman Scient. Tech. 1993).

[7] Arous, G.B. & Léandre, R. Décroissance exponentielle du noyau de la chaleur sur la diagonale (II). Probab. Theory Relat. Fields **90**, 377-402 (1991).

[8] Stroock, D.W. & Varadhan, S.R.S. On the support of diffusion processes with applications to the strong maximum principle in *Proc. Sixth Berkeley Symposium on Mathematical Statistics and Probability, vol. III* 333-359 (University of California Press, Berkeley, 1972).

[9] Xi, F. On the stability of jump-diffusions with Markovian switching. J. Math. Anal. Appl. **341**, 588-600 (2008).

[10] Lasota, A. & Mackey, M.C. Chaos, fractals and noise in *Stochastic aspects of dynamics* 97 (Springer applied mathematical sciences, New York, 1994).

[11] Rudnicki, R. Long-time behaviour of a stochastic prey-predator model. Stoch. Process. Appl. **108**, 93-107 (2003).

[12] Khasminskii, R.Z., Zhu, C. & Yin, G. Stability of regime-switching diffusions. Stoch. Process. Appl. **117**, 1037-1051 (2007).

[13] Pichór, K. & Rudnicki, R. Stability of Markov semigroups and applications to parabolic systems. J. Math. Anal. Appl. **215**, 56-74 (1997).

[14] Lipster, R. A strong law of large numbers for local martingales. Stochastics **3**, 217-228 (1980).
